# A Survey of NOMA for VLC Systems: Research Challenges and Future Trends

**DOI:** 10.3390/s22041395

**Published:** 2022-02-11

**Authors:** Hesham Sadat, Mohamed Abaza, Ali Mansour, Ayman Alfalou

**Affiliations:** 1 LABSTICC UMR CNRS 6285, ENSTA-Bretagne, 29806 Brest, France; hesham.sadat@ensta-bretagne.org (H.S.); mansour@ieee.org (A.M.); 2 L@bISEN, Equipe LSL, Yncrea Ouest, 20 Rue Cuirasse Bretagne, 29200 Brest, France; ayman.al-falou@isen-ouest.yncrea.fr; 3 Arab Academy for Science, Technology and Maritime Transport, Giza 2033, Egypt

**Keywords:** visible light communication (VLC), power domain nonorthogonal multiple access (PD-NOMA), power allocation, MIMO, security, hybrid VLC/RF

## Abstract

Visible light communication (VLC) has become a promising technology for high data rate communications and an attractive complementary to conventional radio frequency (RF) communication. VLC is a secure, energy efficient and cost-effective technology that exploits the existing infrastructure, particularly in indoor environments, for wireless data transmission. Nevertheless, the main limitation of developing high data rate VLC links is the narrow modulation bandwidth of light-emitting diodes (LEDs), which is in the megahertz range. The power domain nonorthogonal multiple access (PD-NOMA) scheme is envisioned to address several challenges in VLC systems. In this paper, we present a detailed overview of PD-NOMA based VLC systems. Moreover, we introduce insights on some PD-NOMA VLC system constraints and challenges such as power allocation, clipping effect, MIMO and security. Finally, we provide open research problems as well as possible directions for future research to pave the way for the implementation of PD-NOMA VLC systems.

## 1. Introduction

**Figure 1:**
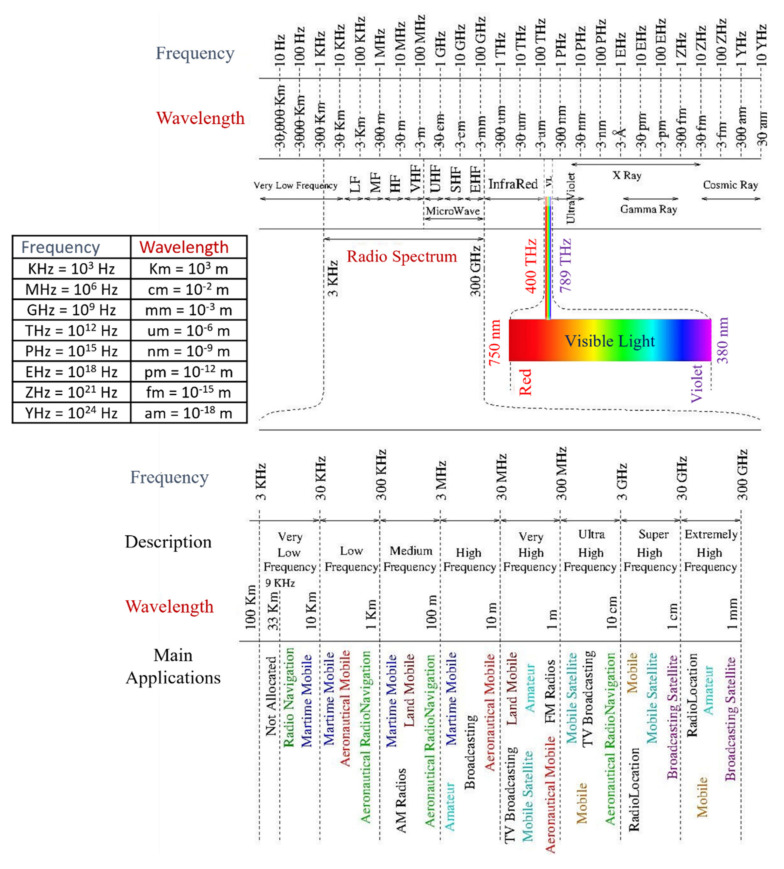
Spectrum allocations, according to their wavelengths λ, frequencies F and applications. This figure is based on an initial figure shown in our previous work [3].

The exponential increase of multimedia applications and wireless connected devices, due to the growth of mobile applications and the advent of the internet of things (IoT), produce an extraordinary growth in traffic demand which requires high-data-rate wireless connectivity [1,2]. In this respect, the next generations (beyond 5G) of wireless networks are expected to provide high system capacity, high quality of service (QoS), massive device connectivity, low latency, and high energy efficiency. Unfortunately, radio frequency (RF) communication in wireless networks suffers from limited spectrum resources, where most applications and services are congested in traditional RF bandwidth sub 6.5 GHz as illustrated in Figure 1 [3]. Moreover, Figure 1 highlights the wideness of the electromagnetic spectrum that can be utilized effectively.

Several features and technologies have opened up many possibilities to fulfill the anticipated requirement of 5G and beyond wireless networks [4]. Some enabling techniques, such as advanced multiple access techniques, new modulation schemes, and massive multiple-input multiple-output (MIMO), can improve the spectral efficiency of the conventional RF communication [5]. Another approach is to utilize the whole RF bandwidth, which extends from 3 kHz to 300 GHz, by exploiting the wide spectrum of millimeter-wave (mmWave) bands (20–100 GHz) to solve the contradiction between capacity requirements and spectrum shortage [5]. However, the major challenges of mmWave communications are its sensitivity to the blockages due to its poor diffracting ability and the enormous propagation loss owing to high carrier frequency [6].

Spectrum extension by utilizing the unoccupied frequency bands is a direct solution for wireless mobile networks, where the global mobile data traffic in 2030 is expected to be up to 5 zettabytes [7,8]. The frequency band (95 GHz–3 THz) has been assigned for the next wireless technology sixth generation (6G) research to fulfill the huge demands for future data traffic according to Federal Communications Commission (FCC) [9,10]. Nevertheless, one of the main technical problems of deploying 6G systems is to overcome the high propagation and atmospheric absorption of THz frequencies, which require a new design for the transceiver [11,12].

**Figure 2:**
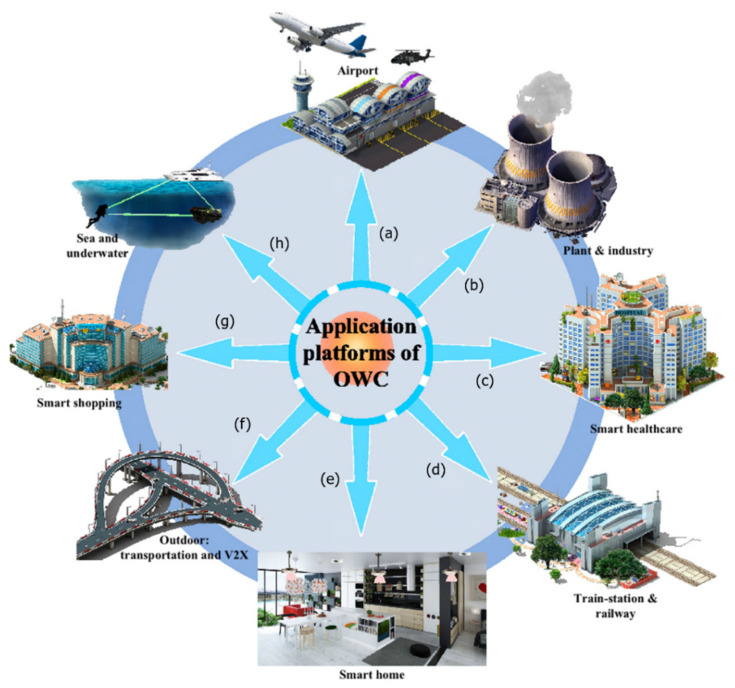
Different OWC technologies used in several applications. (**a**) VLC, (**b**) VLC and FSO, (**c**) VLC, (**d**) VLC and FSO, (**e**) VLC and IR (**f**) VLC and FSO, (**g**) VLC, (**h**) FSO. Ref. [16].

Many researchers recently consider optical wireless communication (OWC) including ultraviolet (UV), infrared (IR) and visible light (VL) as an attractive complementary and alternative technique of RF. OWC systems include outdoor systems, such as free-space optical (FSO), ultraviolet communication (UVC) and underwater communications [13,14]. Moreover, IR communication and visible light communication (VLC) systems are widely used indoor systems [15]. Several OWC applications, utilizing the aforementioned technologies, can be applied to different areas, such as space, industry, healthcare, underwater, indoor public or private places, such as shopping malls, offices and homes, outdoor public places like railway stations and the transportation sector, as shown in Figure 2. Different types of communications are used according to considered the applications such as machine to machine, vehicle to infrastructure or vice-versa, vehicle to vehicle, etc. [16]. It is worth mentioning that VLC can be used in most of these applications.

This survey focuses on VLC as an emergent wireless communication technology that has been proposed as a promising candidate for high-speed communications. Exploring the potential of the license-free visible light band (380–750 nm, i.e., 400–789 THz) of the electromagnetic spectrum, which offers tremendous bandwidth (400 THz), as shown in Figure 1, can solve the RF spectrum congestion and scarcity issues [3]. Moreover, VLC technology has various unique advantages, as follows:

- **Health and safety:** Wavelengths corresponding to VL frequencies are safe to the human body, which makes it possible to transmit with high power in some applications, compared to RF and IR which have power constraints for body and eyes safety [17].
- **Electromagnetic interference (EMI):** The invulnerability of VLC to RF interference makes it suitable for places that are susceptible to EMI as hospitals, aircrafts, mines and petrochemical plants [17].
- **Security and Quality of Service (QoS):** VLC mainly requires a line of sight (LOS) communication, the nature of VL does not penetrate walls that provide small cells with high spatial reuse, high QoS and a secure system that hide data from potential eavesdroppers [17,18].
- **Compatibility with other technologies:** VLC is not supposed to replace RF, but rather complement it where VLC networks can be connected to the existing optical fiber networks and integrated as part of 5G wireless communication systems [17,18].

**Figure 3:**
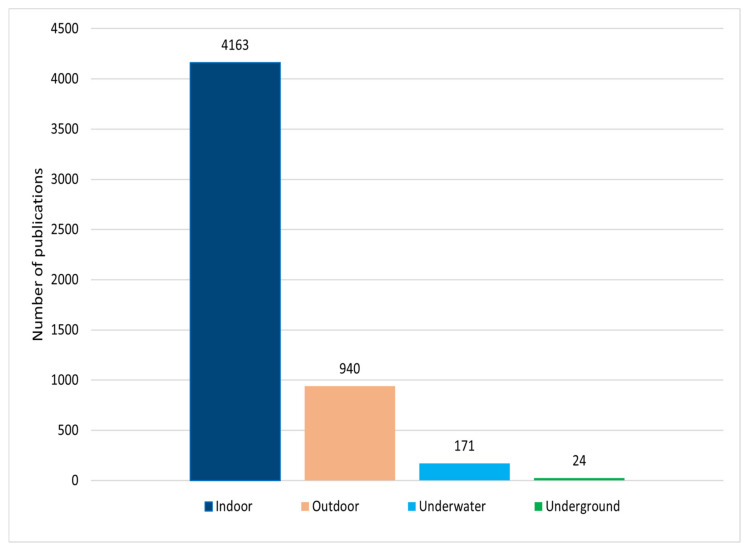
Number of publications on different VLC environments from several journals (IEEE, MDPI, Elsevier and OSA) from 2003 to 2021.

VLC can be exploited in different communication environments, such as indoors, outdoors, underwater, and underground. We have conducted extensive research on visible light communications and Figure 3 presents the number of VLC publications on different environments from various highly reputed journals such as IEEE Xplore (https://ieeeplore.ieee.org/, accessed on 3 December 2021), MDPI (https://www.mdpi.com/, accessed on 3 December 2021), ScienceDirect-Elsevier (https://www.elsevier.com/, accessed on 3 December 2021) and OSA (https://www.osapublishing.org/, accessed on 3 December 2021). It is worth mentioning that around 79% of the research is focused on indoor environments. Therefore, in this survey, we mainly discuss the indoor VLC system models, challenges, and solutions.

**Figure 4:**
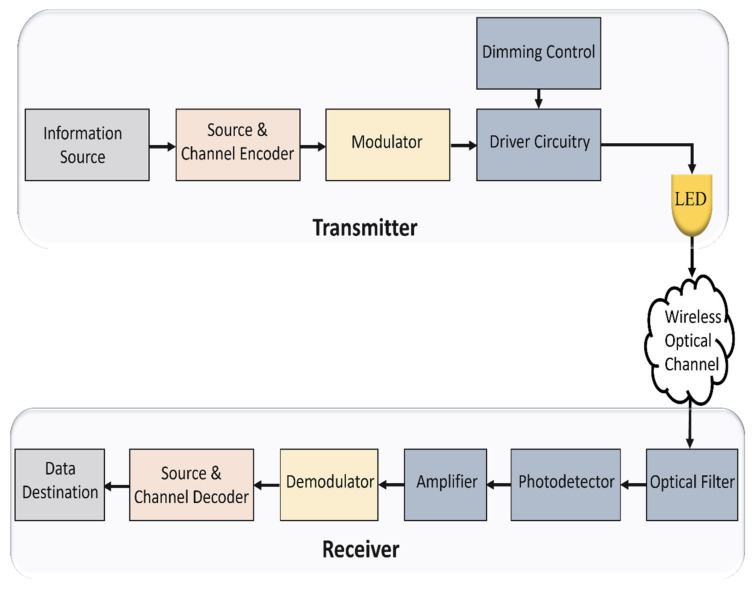
VLC system model.

VLC is a green and energy-efficient technology that exploits the ubiquity of light-emitting diodes (LEDs) lighting infrastructure to provide illumination and, simultaneously, data transmission even when the illuminating light is dimmed or turned off [19]. Typical VLC systems use relatively simple and off-the-shelf components, LED as a transmitter and photodetector (PD) as a receiver, constitute inexpensive systems [18]. The block diagram of the end-to-end VLC system is illustrated in Figure 4. In a process called intensity modulation (IM), LEDs can transmit data by varying the light intensity at a very high frequency without being observed by the human eye [2]. Photodetectors or image sensors at the receiver end, in a process known as direct detection (DD), are used to generate an electrical current proportional to the variation in the received optical power [20].

VLC technology is still in the introductory phase and needs considerable efforts to be widely deployed for practical applications. Nevertheless, various applications are envisioned to be applied on a large scale in the next few years in several research areas: from underwater communication to military purposes, including broadband access, intelligent transportation systems (ITS), power line communication (PLC) and indoor localization [20,21].

**Figure 5:**
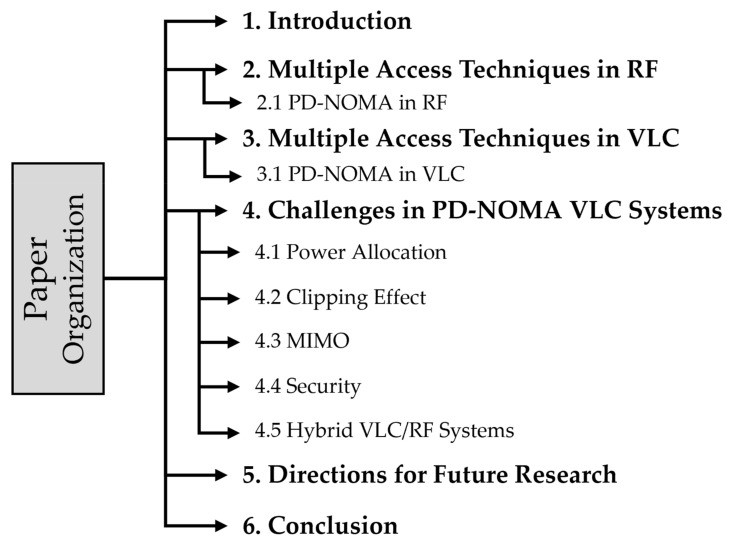
The structure of this paper.

In spite of the aforementioned unique features, VLC systems have certain shortcomings that need to be addressed to utilize the full potential of this emerging technology [2]. The major limitation to developing VLC systems with high achievable data rates is the narrow modulation bandwidths of LEDs (few MHz), which is technically a hardware issue. The commonly white-light LEDs used for VLC are blue LED with yellow phosphorus and RGB LED with modulation bandwidth (2–5 MHz) and (10–20 MHz), respectively [22]. The blue LED-based device, in contrast to RGB LED, can be easily used to achieve better cost effective and energy efficiency; therefore, it becomes more popular in illumination systems [23]. On the other hand, the ability of RGB to provide higher modulation bandwidth makes it more suitable for communication. However, to overcome the modulation bandwidth issue effectively and realize the anticipated full potential of VLC systems, efficient development of high-order modulation techniques, frequency reuse, MIMO, and advanced multiple access schemes are proposed [24]. The structure of the survey paper is depicted in Figure 5. Table 1 shows the list of abbreviations used in this manuscript.

The remainder of this paper is organized as follows: Section 2 provides a brief description of multiple access techniques in RF, followed by a description of the power domain nonorthogonal multiple access (PD-NOMA) scheme. In Section 3, we discuss different multiple access schemes in VLC and present the PD-NOMA-based VLC system model. Section 4 summarizes the survey of the challenges of PD-NOMA-based VLC system including power allocation, clipping effect, MIMO, etc. In Section 5, we highlight the directions for future research. Finally, we conclude the paper in Section 6.

## 2. Multiple Access Techniques in RF

Multiple access techniques used in VLC systems are adopted from their counterparts in RF communication systems [2]. Several orthogonal and nonorthogonal RF multiple access techniques have demonstrated the effective sharing of network valuable resources among a large number of users. In first generation (1G) cellular systems, the technique of dividing the available frequency bandwidth among users known as frequency division multiple access (FDMA) was used to support multiple users. Second generation (2G) cellular technology was based on time division multiple access (TDMA) which allows multiple users to share the same frequency band by dividing the band into different time slots [25]. On the contrary, the code division multiple access (CDMA) technique allowed to allocate frequencies and time intervals to different users with a distinct code to prevent the resulting interference was adopted in third generation (3G) cellular technology. In fourth generation (4G) networks, different frequency subcarriers are assigned to the different users using orthogonal frequency division multiple access (OFDMA) [26].

It is noted that, in the aforementioned orthogonal multiple access (OMA) techniques different users are allocated to orthogonal resources (frequency, time and code). However, 5G networks and beyond need to provide services for a large number of high-data-rate users [24,27]. In contrast to OMA, nonorthogonal multiple access (NOMA) allowed multiple users to share the same frequency band at the same time, and thereby efficiently increasing the system’s spectral efficiency. NOMA is constituted of two main types, namely, PD-NOMA and code domain NOMA (CD-NOMA) [28]. CD-NOMA has the same main operating concept as CDMA, where multiple users can share the entire resources (frequency, time). Nevertheless, CD-NOMA exploits sparse spreading or nonorthogonal low cross-correlation sequences for more users [29]. CD-NOMA comprises several classes, such as sparse code multiple access (SCMA), low-density spreading CDMA (LDS-CDMA), LDS using OFDM (LDS-OFDM). Interested readers are referred to see [30,31,32,33] for greater insights related to CD-NOMA. Some NOMA techniques are multiplexing in different domains including pattern division multiple access (PDMA), lattice partition multiple access (LPMA), building block sparse-constellation-based OMA (BOMA) and spatial division multiple access (SDMA) [34].

**Figure 6:**
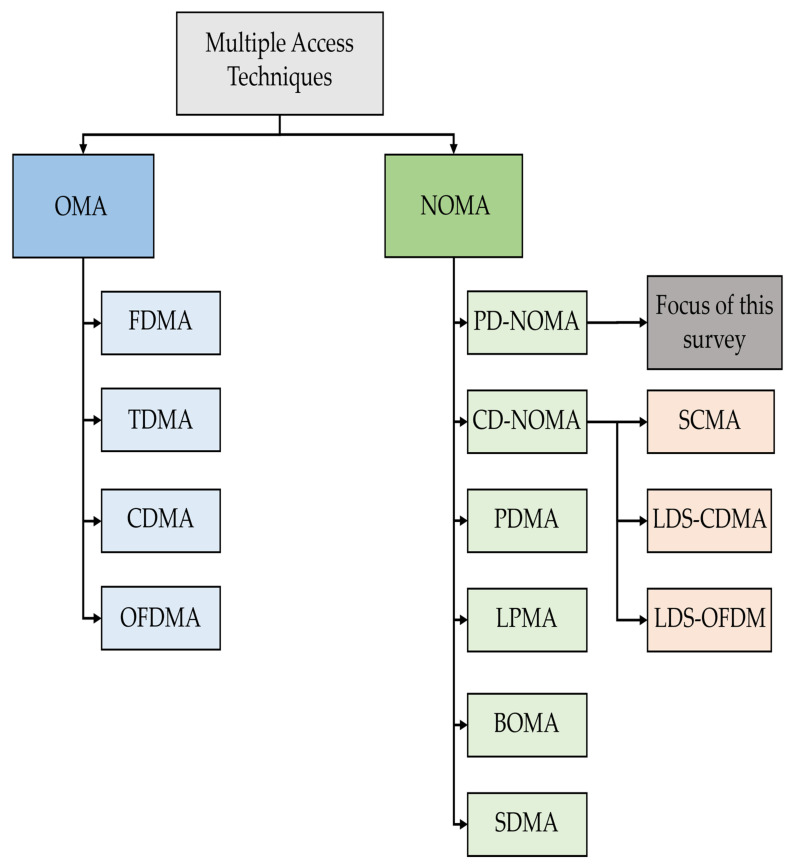
Classification of multiple access techniques.

However, the widely adopted PD-NOMA-based system, which is less complex than the CD-NOMA-based system, is a promising candidate for beyond 5G networks, where multiple users transmit and receive over the entire available frequency and time resources using different power levels [35,36]. Therefore, this survey focuses on PD-NOMA as an attractive technique, for emerging RF and VLC technologies, that have the potential to provide ubiquitous connectivity and high system throughput. Figure 6 presents the classification of multiple access techniques.

### PD-NOMA in RF

**Figure 7:**
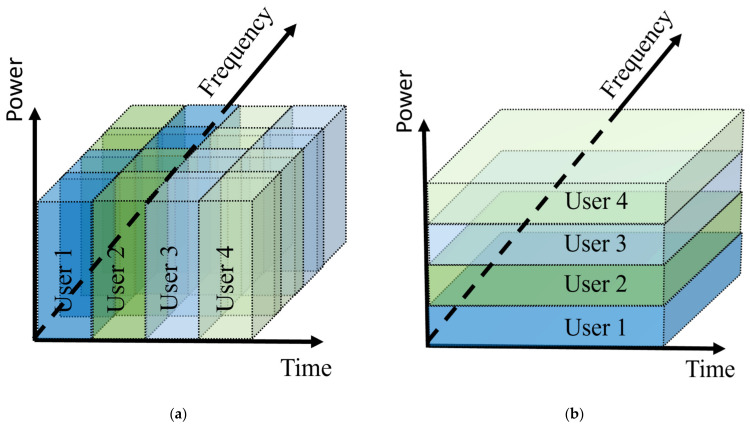
Spectrum sharing for (**a**) OFDMA; (**b**) NOMA.

PD-NOMA as a multiple access technique efficiently utilizes the power domain to serve multiple users simultaneously in the same frequency band and time slot using the same code. To guarantee fairness and effective resources distribution, PD-NOMA allocates higher power to users with a lower signal to noise ratio (SNR) [37]. Unlike OFDMA that sustains the transmitted power of all users at a certain level and divides the available resource blocks (RBs), each user in PD-NOMA has a different power level while all users share the available RBs. Therefore, for the sake of the orthogonality, OFDMA has frequency constrain on subcarrier separation while PD-NOMA utilizes the whole bandwidth without separations [38]. Figure 7 demonstrates the spectrum sharing and power allocation for OFDMA and NOMA.

**Figure 8:**
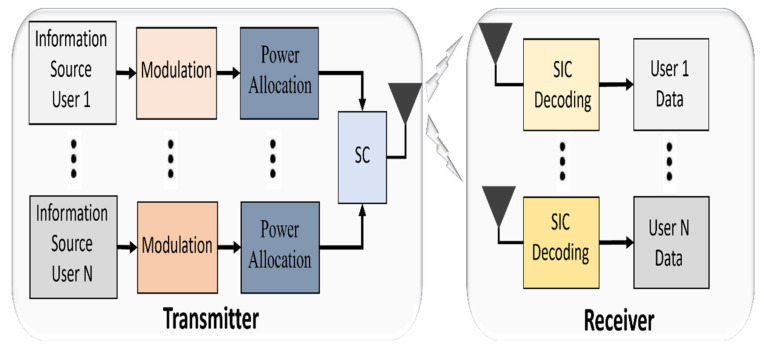
Block diagram of PD-NOMA in the RF system.

At the transmitter end, superposition coding (SC) is applied while a multiuser detection algorithm such as successive interference cancellation (SIC) is performed at the receiver end. Figure 8 illustrates the basic block diagram of PD- NOMA in the RF system. Every user is required to send a pilot sequence for the channel estimation where the power allocation mainly depends on the channel condition which affects the SIC performance. The concept of SC and SIC are discussed in more detail in the next section.

## 3. Multiple Access Techniques in VLC

As aforementioned, the multiple access techniques that are applied on VLC mainly depend on RF ones. Several OMA techniques are studied in VLC systems such as optical TDMA (OTDMA), optical CDMA (OCDMA), wavelength division multiple access (WDMA) and OFDMA. OTDMA in VLC systems requires accurate synchronization between LEDs as access points (APs) and users’ receivers. OTDMA suffers from inter-cell interference (ICI) in multicell scenarios at overlapping areas between the cells, which leads to severe degradation in VLC system performance [39]. The main limitation in OCDMA is to handle a large number of users by generating long optical orthogonal codes (OOC), which increases the complexity of the system and degrades the achievable data rate [40]. In WDMA, multicolor LEDs are used to assign noninterfering wavelengths to users to transmit simultaneously which leads to relative complexity to implement such dense WDMA systems using off-the-shelf LEDs [41]. Among these OMA techniques, researchers have focused on OFDMA as an efficient scheme for VLC networks due to its distinct advantages such as low implementation complexity and high spectral efficiency.

In addition to OMA, different NOMA techniques are applied to VLC systems. Optical SDMA (OSDMA) is the NOMA technique that uses multiple directional optical beams at the transmitter to separate users in the space domain. However, OSDMA increases the implementation complexity of the system due to the design modifications needed in LEDs and optics [42]. Moreover, optical PD-NOMA has recently gained much attention as a promising technique that is able to address the key challenges in VLC systems and outperforms conventional multiple access techniques.

### PD-NOMA in VLC

**Figure 9:**
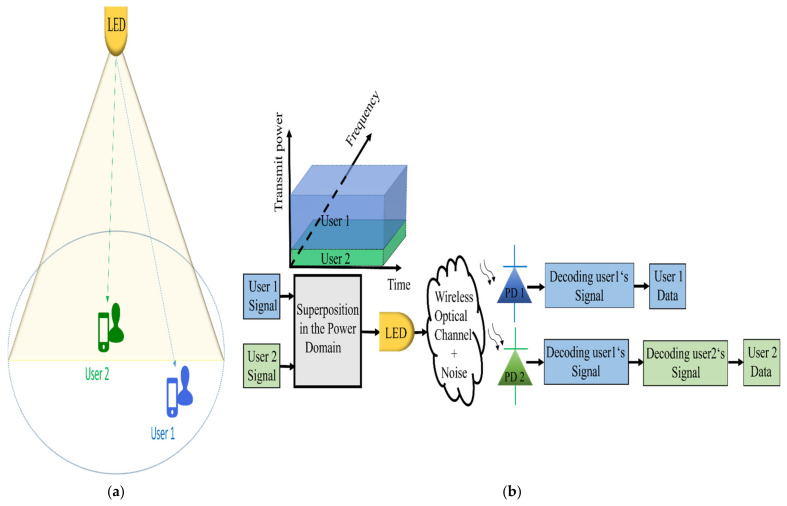
An illustration of a two-user downlink PD-NOMA scheme in the VLC: (**a**) system model; (**b**) SC and SIC.

The process of simultaneous communication of information from one transmitter to different receivers in a downlink broadcast channel is called SC [43]. The main concept of a two-user PD-NOMA in a VLC system with a SC at the transmitter and SIC at the receivers is shown in Figure 9. The transmitter applies SC on the signals of both users and performs a comparison between the channel gains of the users to determine the required power coefficients. User 1, who has a weak received signal, is farther away from the LED (weak user with worse channel gain) and user 2, who has a strong received signal, is close to the LED (strong user with better channel gain) [44,45]. The transmitter assigns higher power to the weak user’s signal *x*_1_, as compared to the strong user’s signal *x*_2_, then superimpose both signals and convey them simultaneously. The superimposed signal *s* can be expressed as:

(1) $$s=P_{1}x_{1}+P_{2}x_{2}$$

where *P*_1_ and *P*_2_ are the power allocation for user 1 and user 2, respectively. The sum of *P*_1_ and *P*_2_ is equal to the total transmitting power. The concept of SC can be applied for a large number of users and the power allocation is assigned based on the channel gain of the user.

PD-NOMA applies SIC at the receiver end for detection. User 1 can directly detect its message from the superimposed received signal and treats the interference from user 2′s signal as noise. On the other hand, user 2 has to apply SIC by detecting user 1′s signal first and subtracting it from the received signal then detecting its signal. For a large number of users, each user (except the farthest user) needs to apply SIC on all stronger signals before it detects its own signal [46].

PD-NOMA scheme offers several benefits, such as achieving user fairness, higher cell-edge throughput, improving spectral efficiency, and low transmission latency (no scheduling request from users to base station is required) [47]. Furthermore, the unique characteristics of VLC networks enhance the PD-NOMA performance as compared to RF for the following reasons:

1. PD-NOMA performance is enhanced at a high signal to noise ratio (SNR), which is the case in VLC networks that consists of small cells that provide short propagation distance and strong LOS. PD-NOMA is efficient in multiplexing a small number of users, which is an advantage in indoor VLC systems [48];
2. Achieving accurate channel state information (CSI) at the transmitter can enhance the power allocation of each user in PD-NOMA. The quasistatic nature of the channel due to low mobility in the indoor VLC systems leads to a more reliable estimation of the CSI [49];
3. Tuning the semiangles of the LEDs and the fields of view (FOVs) of PD can improve PD-NOMA performance in VLC by controlling the channel gain difference between users [50].

PD-NOMA and OFDMA are the most relevant NOMA and OMA schemes for a high data rate in VLC systems, respectively. Therefore, we provide a comparison of their performance in VLC systems. For NOMA, the achievable data rates, assuming frequency-flat channel, for user 1 and user 2 is given as in [51] (here, we consider that the first user is much close to the transmitter than the second one):

(2) $$R_{1}=\frac{B}{2}\;\log_{2}\left(1+\frac{\gamma^{2}L^{2}P\;K_{n}G_{1}^{2}}{\epsilon \;\gamma^{2}L^{2}P^{2}\;(1-K_{n})\;G_{1}^{2}+N_{o}B}\right)$$

(3) $$R_{2}=\frac{B}{2}\;\log_{2}\left(1+\frac{\gamma^{2}L^{2}P\;(1-K_{n})\;G_{2}^{2}}{\gamma^{2}L^{2}P^{2}\;K_{n}G_{2}^{2}+N_{o}B}\right)$$

where *B* is the transmission bandwidth, *γ* is the responsivity, *L* stands for the number of LED chips, *P* becomes the total transmitted power, *K_n_* is the power allocation factor, *G* is the electrical channel DC gain, ϵ is the cancellation error term, *N_o_* is the noise variance. For OFDMA, the achievable data rates for user 1 and user 2 are given as in [51]:

(4) $$R_{1}=\frac{\alpha B}{2}\;\left(1+\frac{\gamma^{2}L^{2}P\;K_{o}G_{1}^{2}}{\alpha BN_{o}}\right)$$

(5) $$R_{2}=\frac{\left(1-\alpha \right)B}{2}\;\left(1+\frac{\gamma^{2}L^{2}P\;(1-K_{o})\;G_{2}^{2}}{\left(1-\alpha \right)BN_{o}}\right)$$

where α and *K_o_* are the allocation coefficients for the total bandwidth B and power, respectively. The 1/2 terms in the above equations indicate the spectral efficiency is lost approximately due to the Hermitian symmetry. In [51], the results showed that the performance of PD-NOMA outperforms OFDMA in terms of the achievable data rate, under perfect interference cancellation, in an indoor downlink VLC system with illumination constraints. On the contrary, when the interference cancellation becomes imperfect the PD-NOMA performance degrades based on the cancellation error percentage. In addition, the authors in [52] derived an analytical expression for the symbol error rate (SER) of PD-NOMA-VLC. The simulation results confirmed the superior performance of PD-NOMA compared to OFDMA in terms of SER. From a large-space indoor scenario standpoint, the sum rate of the multicell VLC network was investigated in [39] for both PD-NOMA and OFDMA. Results showed that PD-NOMA outperforms OFDMA in terms of the total network sum rate for three different complexity scenarios. comparing NOMA with TDMA in [53] shows the ability of NOMA to increase the system capacity by 125% when the semiangle of the LED is 30°. PD-NOMA not only outperforms OFDMA in terms of achievable data rate and system capacity but also system fairness [54].

Consequently, it is evident that PD-NOMA has a superior performance that is able to outperform other multiple access techniques. These distinct advantages offered by PD-NOMA led many researchers to further investigate the performance of PD-NOMA in VLC networks.

## 4. Challenges in PD-NOMA VLC

PD-NOMA can provide useful solutions to several challenges in VLC systems. Nevertheless, applying PD-NOMA to VLC systems creates new constraints and challenges that require effective solutions. We present a detailed overview of some of these challenges and solutions in this section.

### 4.1. Power Allocation

As already mentioned, the PD-NOMA concept is based on assigning a high amount of power to the users with the worse channel gains and a low amount of power to the users with the better channel gains. Consequently, choosing an effective power allocation technique is mandatory to assign a suitable power level to each user for enhancing PD-NOMA performance [44,55]. Different power allocation techniques are proposed for PD-NOMA VLC systems, such as fixed power allocation (FPA), gain ratio power allocation (GRPA), and normalized gain difference power allocation (NGDPA). FPA is a simple power allocation strategy that does not require the exact values of the channel gains to assign power levels to the users [56]. It sorts the users according to their channel gains, which mainly depend on the distance between users and LED, and allocates power based on the decoding order. In [50], a novel GRPA was proposed to ensure fairness as the power allocation not only depends on the decoding order but also the ratio between the user channel gain and the first sorted user gain. Results indicated that GRPA significantly enhanced the system performance in terms of the bit error rate (BER) compared to FPA. NGDPA was proposed in [57] as an efficient with low computational complexity power allocation strategy in indoor MIMO-PD-NOMA VLC systems. The sum rate of NGDPA can achieve up to 29.1% enhancement than GRPA in 2 × 2 MIMO-PD-NOMA VLC systems with three users. Authors in [58] proposed a power allocation scheme called simplified gain ratio power allocation (S-GRPA). S-GRPA is a low complexity power allocation scheme that uses the look-up table method to obtain the CSI. Some optimization techniques are applied to the power allocation schemes to enhance the overall system performance are summarized in Table 2 (all references in the next tables are sorted in a chronological order).

### 4.2. Clipping Effect

In the context of VLC systems, several intensity modulation techniques were commonly used such as on-off keying (OOK), M-ary pulse position modulation (M-PPM) and M-ary pulse amplitude modulation (M-PAM) [26]. Nevertheless, the inter-symbol interference (ISI), due to the demand for a high data rate with a limited modulation bandwidth of LEDs, leads to system performance degradation. Orthogonal frequency division multiplexing (OFDM) is proposed as an efficient solution with a low implementation complexity for ISI [69]. However, the waveforms generated by conventional OFDM (complex and bipolar) require modifications to be compatible with IM/DD systems that deal with real and non-negative waveforms. A real valued OFDM signal can be obtained by using Hermitian symmetry [70]. Moreover, several optical OFDM schemes were introduced to obtain positive valued symbols such as direct current biased optical OFDM (DCO-OFDM) and asymmetrically clipped optical OFDM (ACO-OFDM) [71].

**Figure 10:**
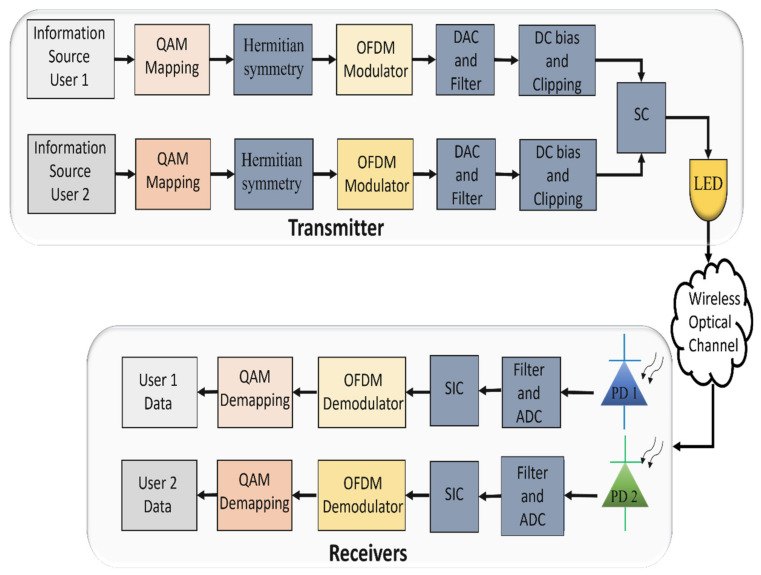
Block diagram of a two-user downlink DCO-OFDM-based PD-NOMA-VLC system.

The block diagram of a DCO-OFDM-based PD-NOMA transceiver is shown in Figure 10. DCO-OFDM technique is commonly used to shift the signal by adding DC bias to obtain a unipolar signal. Weiwen et al. [72] incorporated the PD-NOMA with DCO-OFDM to increase the system’s spectral efficiency. the effect of clipping noise and attenuation factor through double-sided asymmetrical clipping have been studied. Moreover, the analytical and simulation results showed that PD-NOMA DCO-OFDM outperforms OMA DCO-OFDM in terms of achievable rate regions. In [73], the authors proposed a hierarchical predistorted layered asymmetrically clipped optical OFDM (HPD-LACO-OFDM) scheme for PD-NOMA VLC networks to improve the optical power efficiency of DCO-OFDM while keeping its high spectrum efficiency. Furthermore, their experimental results showed the superior BER performance of the HPD-LACO-OFDM-based PD-NOMA-VLC network with three layers over the traditional DCO-OFDM. The authors in [74] investigated the impact of the clipping noise of polarity divided DCO-OFDM (PD-DCO-OFDM) and DCO-OFDM-based PD-NOMA-VLC system, on the achievable sum rate. Their simulation results showed that PD-DCO-OFDM can offer a better achievable sum rate compared to DCO-OFDM for the two-user scenario. In [75], the authors proposed zero-biased PD-NOMA for VLC systems. It is found that the zero-biased PD-NOMA enhances the system performance compared to the other OFDM schemes in PD-NOMA VLC systems. Furthermore, zero-biased PD-NOMA offers a better achievable data rate, spectral efficiency and energy efficiency than zero-biased OFDMA technique. The IEEE 802.15.13 standard target is to deliver high data rates up to 10 Gbit/sec at 200 m range and is expected to use DCO-OFDM. Adopting MIMO techniques in combining with DCO-OFDM would help to achieve such ambitious data rates [26].

### 4.3. MIMO

**Figure 11:**
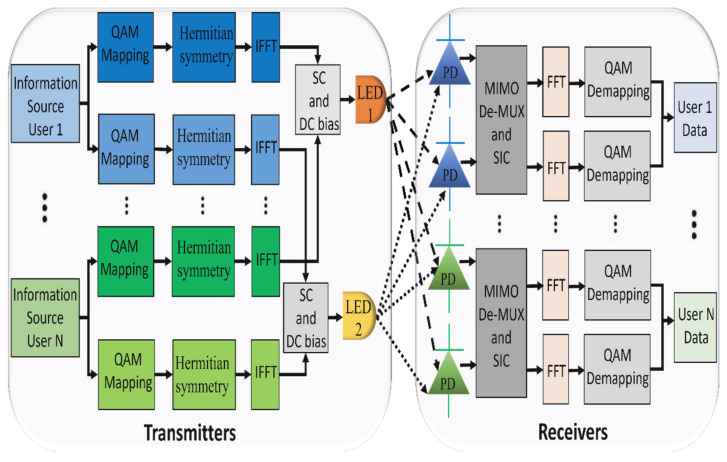
Block diagram of 2 × 2 MIMO-PD-NOMA-based VLC system with N users.

Most indoor environments use multiple LEDs to provide sufficient illumination, which motivates the implementation of MIMO in VLC systems. Employing MIMO techniques in VLC systems can increase spectral efficiency by overcoming the narrow modulation bandwidth limitation of the LEDs [76,77]. The block diagram of the 2 × 2 MIMO PD-NOMA-based VLC system is shown in Figure 11. Spatial multiplexing can be used with MIMO by splitting the users into groups and each group served by a specific LED. Users within the same group, utilize the overall modulation bandwidth, are superimposed in the power domain [78]. Precoding schemes are applied in multiuser MIMO-VLC systems to separate the received signals for the MIMO subchannels [79,80]. Moreover, SIC can be performed to eliminate the interference between the multiplexed users within the same group. Exploit MIMO-VLC can substantially enhance the system capacity by serving a large number of users simultaneously [81]. However, the performance of PD-NOMA mainly depends on the adopted power allocation technique and user grouping strategy, which increases the design complexity for MIMO-PD-NOMA-based VLC systems. The summary of MIMO-PD-NOMA-based VLC systems is provided in Table 3.

### 4.4. Security

VLC channels characteristics are distinctive compared to RF ones. As already mentioned, VLC networks are more secure than RF networks, as the light cannot penetrate walls, which protect its wireless communication from the outdoor eavesdroppers [89]. Nevertheless, securing VLC networks is required, due to the broadcast nature of VLC, where the signals can be available for eavesdroppers in public areas such as libraries and malls or multiple-user indoor scenarios such as meeting rooms [90]. The upper layers are usually responsible for securing the communication by exploiting the encryption methods, while the research in physical layers is related to providing reliable signal transmission [91].

However, a promising complementary technique, due to the need for higher data rates, is proposed to enhance the security of wireless communication using a physical layer [92]. The physical layer security (PLS) is an alternative way to achieve secure communication without encryption by utilizing the wireless medium characteristics [93]. The wireless channel disadvantages as the randomness of noise, fading and different transmitters such as multi-LED or cooperative relays are harnessed from PLS to ensure a secure transmission, by distinguishing between the SNR of legitimate user and eavesdropper, which reduces the extracted information by the eavesdropper [94,95].

In PD-NOMA-VLC networks the users share the same resources (frequency and time) which increase the need for PLS to improve the network information security. The results of the previous works on the PLS in RF cannot be directly applied on VLC, due to the channel capacity difference between both technologies, where the issue of the nonlinear distortion on VLC systems and positive real valued signal requirements [96]. Recently, research on PLS for PD-NOMA-VLC networks using different techniques, such as beamforming and artificial noise (AN) has attracted great interest [97]. In [98], the authors investigated the PLS in a multiuser PD-NOMA-VLC for both single- and multieavesdroppers. The security performance is evaluated by deriving an expression for secrecy outage probability (SOP) in the case of a single eavesdropper. Moreover, according to the spatial distribution of the users, the SOP is obtained in the presence of multieavesdroppers. Xiang and Jinyong [99] studied the PLS of the strong user in a multiple-input single-output (MISO) PD-NOMA-VLC system that consists of two users and an eavesdropper. The authors compared the SOP performance of the MISO PD-NOMA-VLC system with the single-input single-output (SISO) PD-NOMA-VLC system and the results showed that the superiority of the MISO PD-NOMA-VLC system. Authors in [96] investigated resource allocation to minimize the transmitted power of the MISO PD-NOMA-VLC system and introduced AN jamming and beamforming to improve the system performance and provide secure communication. The optimal security beamforming design to minimize the transmitted power under dimming control and the achievable security rate were investigated in [90]. In addition to PLS, the authors of [100] implemented a two-level chaotic encryption algorithm in the PD-NOMA-VLC system to provide both security (against eavesdropper) and privacy (among the legitimate users).

### 4.5. Hybrid VLC/RF Systems

According to [101], around 80% of the wireless data traffic originates from indoor applications. Consequently, VLC has been envisioned as an alternative/complementary technology that offers several advantages over RF, especially in indoor environments, such as exploiting the existed infrastructure for illumination to design a low cost system that offers ultra-high data rate [102]. In spite of the unique advantages of VLC, several drawbacks that can degrade the VLC system performance significantly have to be taken into consideration. The main challenge of VLC is the severe performance degradation in the absence of LOS communication which is not the case in RF, which can exploit the potential NLOS communication effectively [103]. In addition, unlike downlink, the uplink transmission scenario in VLC systems is not efficient due to the unwanted irradiance. In this context, a promising direction to overcome these drawbacks is developing a hybrid VLC/RF heterogeneous network (HetNet) that is able to combine the advantages of both VLC and RF systems and enhance users’ mobility as well as provide ubiquitous coverage [104,105]. Recently, hybrid VLC/RF networks have attracted considerable attention as an effective solution that can provide performance improvement for indoor communication. Moreover, introducing PD-NOMA to such hybrid networks can increase both connectivity and spectral efficiency. The related PD-NOMA research activities based on hybrid VLC/RF systems are shown in Table 4.

## 5. Directions for Future Research

As discussed in the previous section, the adoption of PD-NOMA in indoor VLC systems has significant benefits that have attracted much research attention. However, this research point is still in the introductory phase as several research areas needed to be covered properly before being able to apply it in different applications. Therefore, we provide insights into some challenges, open research problems and potential solutions as follows:

1. It is obvious that most of the research in PD-NOMA-based indoor VLC systems focused on the downlink. Uplink performance needs further investigation to address the challenges of implementing a full duplex PD-NOMA VLC system. However, few studies discussed this point such as [116] proposed phase predistortion method to decrease the uplink error rate and [117] suggested a joint detection method to improve the BER performance.
2. An LED in indoor environments covers a small area with a limited number of users. In the case of a large number of users, the performance of PD-NOMA will degrade due to the users’ channel conditions similarity. Consequently, adopting hybrid PD-NOMA/OMA is beneficial in this case, where users are multiplexed into groups using OMA scheme as OFDMA and distinguish between users in each group using PD-NOMA.
3. Intracell interference and ICI degrade the PD-NOMA VLC system performance in both uplink and downlink. Therefore, precoding schemes and more advanced power allocation techniques are needed for multicell scenarios.
4. The nonlinearity of the LED is a challenge that has to be investigated to realize the actual performance of the PD-NOMA VLC system [118]. Compensation techniques and mitigation strategies for nonlinear distortions are needed to provide high data rate PD-NOMA VLC systems especially under imperfect CSI.
5. A cooperative PD-NOMA VLC system was proposed in [45] where the near users to the ceiling light act as relays to assist the far users. It is worth studying such systems for mobile users under imperfect CSI to enhance the BER of cell-edge users.
6. Error propagation problem owing to SIC is an issue that can degrade the BER performance of the PD-NOMA VLC system and causes user unfairness [119]. Therefore, adopting advanced modulation techniques are expected to mitigate SIC error propagation.
7. Implementation of PD-NOMA VLC systems needs further investigation of massive MIMO, advanced user grouping techniques and dimming control.
8. The PD-NOMA system performance significantly depends on the power allocation technique [120,121]. Consequently, more practical studies on improving power allocation techniques are required especially in the case of a large number of users.

## 6. Conclusions

In this paper, we presented VLC as a promising wireless technology for indoor communication. Several multiple access techniques in VLC systems including OTDMA, OCDMA, OFDMA, WDMA and OSDMA were reviewed. We discussed, in detail, PD-NOMA as a promising scheme that can overcome the VLC limitations. Furthermore, we reported PD-NOMA’s challenges in VLC systems, such as power allocation, clipping effect, MIMO and security. The directions for future research are also highlighted. Finally, VLC systems with PD-NOMA are expected to contribute effectively to meet the extraordinary traffic demand in the future generation of wireless networks.

## Figures and Tables

**Table 1:** List of abbreviations.

Abbreviations	Description	Abbreviations	Description
1G	First generation	MADM	Multiattribute decision making
2G	Second generation	MFOPA	Multifactor control optimal power allocation
3G	Third generation	MIMO	Multiple-input multiple-output
4G	Fourth generation	MISO	Multiple-input single-output
5G	Fifth generation	MMSE	Minimum mean square error
6G	Sixth generation	mmWave	Millimeter-wave
ACO-OFDM	Asymmetrically clipped optical OFDM	M-PAM	M-ary pulse amplitude modulation
ADC	Analog to digital converter	M-PPM	M-ary pulse position modulation
AN	Artificial noise	NGDPA	Normalized gain difference power allocation
AP	Access point	NLGRPA	Normalized logarithmic gain ratio power Allocation
BD	Block diagonalization	NLOS	Non-LOS
BER	Bit error rate	NOMA	Nonorthogonal multiple access
BMUP	Bipartite matching based user pairing	OCDMA	Optical CDMA
BOMA	Building block sparse constellation based OMA	OFDM	Orthogonal frequency division multiplexing
CBSC	Cross-band selection combining	OFDMA	Orthogonal frequency division multiple access
CDMA	Code division multiple access	OMA	Orthogonal multiple access
CD-NOMA	Code-domain noma	OOC	Optical orthogonal codes
CSI	Channel state information	OOK	On-off keying
DAC	Digital to analog converter	OQAM	Offset QAM
DCO-OFDM	Direct current biased optical OFDM	OSDMA	Optical SDMA
DD	Direct detection	OTDMA	Optical TDMA
DE	Differential evolution	OWC	Optical wireless communication
EMI	Electromagnetic interference	PD	Photodetector
EPA	Enhanced power allocation	PDMA	Pattern division multiple access
FCC	Federal Communications Commission	PD-NOMA	Power-domain noma
FDMA	Frequency division multiple access	PLC	Power line communication
FFT	Fast Fourier transform	PLS	Physical layer security
FOV	Field of view	PS	Power splitting
FPA	Fixed power allocation	QAM	Quadrature amplitude modulation
FSO	Free-space optical	QoS	Quality of service
GP	Gradient projection	RB	Resource block
GRPA	Gain ratio power allocation	RF	Radio frequency
HetNet	Heterogeneous network	SC	Superposition coding
HPV	Hybrid power line VLC	SCMA	Sparse code multiple access
ICI	Intercell interference	SDMA	Space division multiple access
IFFT	Inverse FFT	SER	Symbol error rate
IM	Intensity modulation	S-GRPA	Simplified gain ratio power allocation
IoT	Internet of Things	SIC	Successive interference cancellation
IPA	Inverse power allocation	SISO	Single-input single-output
IR	Infrared	SNR	Signal to noise ratio
ISFA	Intrasymbol frequency averaging	SOP	Secrecy outage probability
ISI	Intersymbol interference	SWIPT	Simultaneous wireless information and power transfer
ITS	Intelligent transportation systems	TDMA	Time division multiple access
KKT	Karush–Kuhn–Tucker	UV	Ultraviolet
LDS-CDMA	Low-density spreading CDMA	UVC	Ultraviolet communication
LDS-OFDM	Low-density spreading OFDM	VL	Visible light
LED	Light-emitting diode	VLC	Visible light communication
LOS	Line of sight	WDMA	Wavelength division multiple access
LPMA	Lattice partition multiple access	ZF	Zero forcing

**Table 2:** A summary of power allocation optimization in PD-NOMA downlink VLC systems.

System Model	Optimization Method	Design Objective	Contribution	Ref.
1 VLC AP + K users	Karush–Kuhn–Tucker (KKT) conditions for optimality	Sum rate	Propose a low complexity power control algorithm that outperforms the conventional OFDM in terms of sum rate	[59]
4 VLC AP + K users	Gradient projection (GP) algorithm	Sum rate + User rate	Investigate a power allocation scheme under QoS constraint and user grouping that achieves a higher sum rate performance than OMA	[60]
1 VLC AP + 2 users	GP algorithm	Sum rate+ Fairness	Propose an optimal power allocation that outperforms the OMA scheme taking into consideration practical optical power and QoS constraints	[61]
4 VLC AP + K users	Analytical method	Sum rate	The sum rate performance of the proposed enhanced power allocation (EPA) algorithm outperforms the optimized FPA and GRPA	[62]
1 VLC AP with N LEDs + K users	Interior-point algorithm	Achievable rate	Propose optimal power allocation schemes for both static and mobile users and derive a closed form expression for achievable rates of static users	[63]
1 VLC AP + K users	Analytical method	Achievable rate	Introduce an adaptive power allocation scheme that chooses between GRPA or inverse power allocation (IPA) and the optimal power allocation factor to increase the achievable rate using multiattribute decision making (MADM)	[64]
1 VLC AP + K users	KKT conditions for optimality	Sum rate	Introduce a joint PLC-VLC power allocation scheme that outperforms FPA and NGDPA in terms of sum rate	[65]
1 VLC AP + K users	Interior-point algorithm	Sum rate	The sum rate performance of the proposed multifactor control optimal power allocation (MFOPA) outperforms FPA and GRPA	[66]
4 VLC AP + K users	KKT conditions for optimality	Sum rate	Present an optimal power allocation for a downlink hybrid power line VLC (HPV) system that maximizes sum rate compared to FPA and GRPA	[67]
1 VLC AP + K users	Differential evolution (DE)-based heuristic algorithm	Sum rate	Verify the superiority of two proposed power allocation schemes over the conventional FPA and GRPA in terms of sum rate and user fairness using simulation	[68]

**Table 3:** A summary of MIMO-PD-NOMA-based VLC systems.

System Model	Design Objective	Contribution	Ref.
2 × 2 MIMO with N users	BER	Evaluate the power allocation ratio for the best BER performance and proposed minimum mean square error (MMSE) and intrasymbol frequency averaging (ISFA) as efficient channel estimation methods to eliminate the interuser interference effectively.	[82]
2 × 2 MIMO with N users	BER	Propose the offset quadrature amplitude modulation (OQAM)- OFDM-based MIMO-NOMA and compared its performance with conventional OQAM-based MIMO-OFDM	[83]
4 × 2 MIMO with N users	Achievable sum rate	Propose the low computational complexity normalized logarithmic gain ratio power allocation (NLGRPA) which outperforms NGDPA and GRPA methods	[84]
2 × 2 MIMO with N users	Achievable sum rate	Propose the low complexity NGDPA method and compared it with GRPA	[85]
2 × 2 MIMO with N users	Sum rate	Evaluate the percentage gain of sum rate for both (LOS) and (LOS + NLOS) in a single reflection environment and calculated the delay spread using NGDPA and GRPA methods	[86]
M × L MIMO with N users	Spectral efficiency	Propose an algorithm for grouping the users into clusters based on the correlation among their channel gains and the proposed algorithm has better performance than zero forcing (ZF) and block diagonalization (BD) precoding schemes	[78]
2 × 2 MIMO with N users	Achievable capacity	Formulate an analytical model for system capacity and evaluate the performance of GRPA and NGDPA and compared them in terms of system coverage, user density and user location	[87]
2 × 2 MIMO with N users	BER	Analyze MMSE equalizer with SIC and results showed it outperforms the ZF equalizer with SIC	[88]

**Table 4:** A summary of PD-NOMA-based hybrid VLC/RF systems.

System Model	PD-NOMA Applied to	Cooperation	Design Objective	Contribution	Ref.
1 VLC AP + K users	VLC	Cooperative system	Outage probability	Propose the bipartite matching-based user pairing (BMUP) scheme to solve optimal user pairing problem and to reduce decoding complexity	[106]
1 VLC AP + 2 users	VLC	Cooperative system	Outage probability	Investigate a cooperative system where the near user can support the far user without needing extra power by utilizing simultaneous wireless information and power transfer (SWIPT) and study the effect of power splitting (PS) ratio on the system performance	[107]
M VLC APs + 1 RF AP + K users	VLC and RF	Cooperative system	Sum rate	Propose coalitional game theory for user grouping where each coalition is allocated to a certain AP either VLC or RF and the user is able to change its coalition according to its payoff	[108]
M VLC APs + 1 RF AP + K users	VLC and RF	Cooperative system	Sum rate	Present an efficient power allocation policy and a coalition formation algorithm for user grouping using coalitional game theory and the proposed system has better performance than the non-cooperative scheme	[109]
M VLC APs + N RF APs + K users	VLC and RF	Non-cooperative system	Energy efficiency	Investigate the energy efficiency performance of PD-NOMA under imperfect CSI which outperforms its OFDMA counterpart and it is more robust to CSI errors and LOS variations	[110]
M VLC APs + N RF APs + K users	VLC and RF	Non-cooperative system	Sum rate + energy efficiency	Derive closed-form expressions for the average sum-rate and average energy efficiency and compare the performance of PD-NOMA-based hybrid VLC/RF with PD-NOMA VLC system	[111]
1 VLC AP + 2 users	VLC	Cooperative system	Outage probability + sum throughput	Introduce a cross-band selection combining (CBSC) method which allows the far user to choose between the mixed VLC/RF and the VLC link.	[112]
M VLC APs + 2 × M users	VLC	Cooperative system	Fairness + sum rate	Solve sum rate maximization problem for a cooperative PD-NOMA scheme under QoS and power constraints and compared its performance to non-cooperative one	[113]
1 VLC AP + K paired users	VLC	Cooperative system	Fairness + sum rate	The authors extend the work in [113] to include an investigation of user pairing	[114]
1 VLC AP + K users	VLC and RF	Cooperative system	Outage probability + reliability	Analyze the outage probability for different power allocation techniques and observed the impact of increasing the target data rate as well as the number of users on the system performance	[115]

## Data Availability

Not applicable.

## References

[1] Agiwal M., Roy A., Saxena N. (2016). Next Generation 5G Wireless Networks: A Comprehensive Survey. IEEE Commun. Surv. Tutor..

[2] Marshoud H., Muhaidat S., Sofotasios P.C., Hussain S., Imran M.A., Sharif B.S. (2018). Optical Non-Orthogonal Multiple Access for Visible Light Communication. IEEE Wirel. Commun..

[3] Mansour A., Mesleh R., Abaza M. (2017). New Challenges in Wireless and Free Space Optical Communications. Opt. Lasers Eng..

[4] Dogra A., Jha R.K., Jain S. (2021). A Survey on Beyond 5G Network with the Advent of 6G: Architecture and Emerging Technologies. IEEE Access.

[5] Zhang X., Liu Y., Wang Y., Bai J. (2019). Performance Analysis and Optimization for Non-Uniformly Deployed MmWave Cellular Network. EURASIP J. Wirel. Com Netw..

[6] Seker C., Guneser M.T., Ozturk T. A Review of Millimeter Wave Communication for 5G. Proceedings of the 2018 2nd International Symposium on Multidisciplinary Studies and Innovative Technologies (ISMSIT).

[7] Saha R.K. (2020). A Hybrid Interweave–Underlay Countrywide Millimeter-Wave Spectrum Access and Reuse Technique for CR Indoor Small Cells in 5G/6G Era. Sensors.

[8] International Telecommunication Union Traffic Estimates for the Years 2020 to 2030. Rep. ITU 2015,2370..

[9] Chen S., Liang Y.-C., Sun S., Kang S., Cheng W., Peng M. (2020). Vision, Requirements, and Technology Trend of 6G: How to Tackle the Challenges of System Coverage, Capacity, User Data-Rate and Movement Speed. IEEE Wirel. Commun..

[10] Xing Y., Kanhere O., Ju S., Rappaport T.S. Indoor Wireless Channel Properties at Millimeter Wave and Sub-Terahertz Frequencies. Proceedings of the 2019 IEEE Global Communications Conference (GLOBECOM).

[11] Al-Saman A., Mohamed M., Cheffena M., Moldsvor A. (2021). Wideband Channel Characterization for 6G Networks in Industrial Environments. Sensors.

[12] Chowdhury M.Z., Shahjalal M.D., Ahmed S., Jang Y.M. (2020). 6G Wireless Communication Systems: Applications, Requirements, Technologies, Challenges, and Research Directions. IEEE Open J. Commun. Soc..

[13] Abaza M., Mesleh R., Mansour A., Alfalou A. MIMO Techniques for High Data Rate Free Space Optical Communication System in Log-Normal Channel. Proceedings of the 2013 the International Conference on Technological Advances in Electrical, Electronics and Computer Engineering (TAEECE).

[14] Abaza M., Mesleh R., Mansour A., Aggoune E.-H. (2015). Performance Analysis of MISO Multi-Hop FSO Links over Log-Normal Channels with Fog and Beam Divergence Attenuations. Opt. Commun..

[15] Uysal M., Nouri H. Optical Wireless Communications—An Emerging Technology. Proceedings of the 2014 16th International Conference on Transparent Optical Networks (ICTON).

[16] Chowdhury M.Z., Hossan M.T., Islam A., Jang Y.M. (2018). A Comparative Survey of Optical Wireless Technologies: Architectures and Applications. IEEE Access.

[17] Kavehrad M. (2007). Broadband Room Service by Light. Sci. Am..

[18] Medina C., Zambrano M., Navarro K. (2015). LED Based Visible Light Communication: Technology, Applications and Challenges—A Survey. Int. J. Adv. Eng. Technol..

[19] Lin B., Guo Q., Ghassemlooy Z., Tang X., Lin C., Zhou Z. (2018). Experimental Demonstration of a Non-Orthogonal Multiple Access Scheme for Visible Light Communications with SCFDM Transmission. Phys. Commun..

[20] Obeed M., Salhab A.M., Alouini M.-S., Zummo S.A. (2019). On Optimizing VLC Networks for Downlink Multi-User Transmission: A Survey. IEEE Commun. Surv. Tutor..

[21] Shaaban K., Shamim M.H.M., Abdur-Rouf K. (2021). Visible Light Communication for Intelligent Transportation Systems: A Review of the Latest Technologies. J. Traffic Transp. Eng..

[22] Mapunda G.A., Ramogomana R., Marata L., Basutli B., Khan A.S., Chuma J.M. (2020). Indoor Visible Light Communication: A Tutorial and Survey. Wirel. Commun. Mob. Comput..

[23] Udvary E. (2019). Visible Light Communication Survey. Infocommunications J..

[24] Mathur H., Deepa T. (2021). A Survey on Advanced Multiple Access Techniques for 5G and Beyond Wireless Communications. Wirel. Pers. Commun..

[25] Bawazir S.S., Sofotasios P.C., Muhaidat S., Al-Hammadi Y., Karagiannidis G.K. (2018). Multiple Access for Visible Light Communications: Research Challenges and Future Trends. IEEE Access.

[26] Al-Ahmadi S., Maraqa O., Uysal M., Sait S.M. (2018). Multi-User Visible Light Communications: State-of-the-Art and Future Directions. IEEE Access.

[27] Ding Z., Peng M., Poor H.V. (2015). Cooperative Non-Orthogonal Multiple Access in 5G Systems. IEEE Commun. Lett..

[28] Akbar A., Jangsher S., Bhatti F.A. (2021). NOMA and 5G Emerging Technologies: A Survey on Issues and Solution Techniques. Comput. Netw..

[29] Anwar A., Seet B.-C., Hasan M.A., Li X.J. (2019). A Survey on Application of Non-Orthogonal Multiple Access to Different Wireless Networks. Electronics.

[30] Nikopour H., Baligh H. Sparse Code Multiple Access. Proceedings of the 2013 IEEE 24th Annual International Symposium on Personal, Indoor, and Mobile Radio Communications (PIMRC).

[31] Hoshyar R., Wathan F.P., Tafazolli R. (2008). Novel Low-Density Signature for Synchronous CDMA Systems Over AWGN Channel. IEEE Trans. Signal Processing.

[32] Razavi R., Hoshyar R., Imran M.A., Wang Y. (2011). Information Theoretic Analysis of LDS Scheme. IEEE Commun. Lett..

[33] Hoshyar R., Razavi R., Al-Imari M. LDS-OFDM an Efficient Multiple Access Technique. Proceedings of the 2010 IEEE 71st Vehicular Technology Conference (VTC).

[34] Reddy B.S.K. (2021). Experimental Validation of Non-Orthogonal Multiple Access (NOMA) Technique Using Software Defined Radio. Wirel. Pers. Commun..

[35] Choi J. NOMA: Principles and Recent Results. Proceedings of the 2017 International Symposium on Wireless Communication Systems (ISWCS).

[36] Moltafet M., Yamchi N.M., Javan M.R., Azmi P. (2018). Comparison Study between PD-NOMA and SCMA. IEEE Trans. Veh. Technol..

[37] Ding Z., Lei X., Karagiannidis G.K., Schober R., Yuan J., Bhargava V.K. (2017). A Survey on Non-Orthogonal Multiple Access for 5G Networks: Research Challenges and Future Trends. IEEE J. Sel. Areas Commun..

[38] Kizilirmak R.C., Bizaki H.K. (2016). Non-Orthogonal Multiple Access (NOMA) for 5G Networks. Towards 5G Wireless Networks—A Physical Layer Perspective.

[39] Eltokhey M.W., Khalighi M.-A., Ghassemlooy Z. Multiple Access Techniques for VLC in Large Space Indoor Scenarios: A Comparative Study. Proceedings of the 2019 15th International Conference on Telecommunications (ConTEL).

[40] Pathak P.H., Feng X., Hu P., Mohapatra P. (2015). Visible Light Communication, Networking, and Sensing: A Survey, Potential and Challenges. IEEE Commun. Surv. Tutor..

[41] Chun H., Rajbhandari S., Faulkner G., Tsonev D., Xie E., McKendry J.J.D., Gu E., Dawson M.D., O’Brien D.C., Haas H. (2016). LED Based Wavelength Division Multiplexed 10 Gb/s Visible Light Communications. J. Lightwave Technol..

[42] Chen Z., Basnayaka D.A., Haas H. (2017). Space Division Multiple Access for Optical Attocell Network Using Angle Diversity Transmitters. J. Lightwave Technol..

[43] Cover T. (1972). Broadcast Channels. IEEE Trans. Inf. Theory.

[44] Marshoud H., Sofotasios P.C., Muhaidat S., Karagiannidis G.K., Sharif B.S. (2017). On the Performance of Visible Light Communication Systems with Non-Orthogonal Multiple Access. IEEE Trans. Wirel. Commun..

[45] Sadat H., Abaza M., Gasser S.M., ElBadawy H. (2019). Performance Analysis of Cooperative Non-Orthogonal Multiple Access in Visible Light Communication. Appl. Sci..

[46] Yin L., Popoola W.O., Wu X., Haas H. (2016). Performance Evaluation of Non-Orthogonal Multiple Access in Visible Light Communication. IEEE Trans. Commun..

[47] Higuchi K., Benjebbour A. (2015). Non-Orthogonal Multiple Access (NOMA) with Successive Interference Cancellation for Future Radio Access. IEICE Trans. Commun..

[48] Ding Z., Yang Z., Fan P., Poor H.V. (2014). On the Performance of Non-Orthogonal Multiple Access in 5G Systems with Randomly Deployed Users. IEEE Signal Processing Lett..

[49] Maraqa O., Rajasekaran A.S., Al-Ahmadi S., Yanikomeroglu H., Sait S.M. (2020). A Survey of Rate-Optimal Power Domain NOMA With Enabling Technologies of Future Wireless Networks. IEEE Commun. Surv. Tutor..

[50] Marshoud H., Kapinas V.M., Karagiannidis G.K., Muhaidat S. (2016). Non-Orthogonal Multiple Access for Visible Light Communications. IEEE Photon. Technol. Lett..

[51] Kizilirmak R.C., Rowell C.R., Uysal M. Non-Orthogonal Multiple Access (NOMA) for Indoor Visible Light Communications. Proceedings of the 4th International Workshop on Optical Wireless Communications (IWOW).

[52] Huang H., Wang J., Wang J., Yang J., Xiong J., Gui G. (2017). Symbol Error Rate Performance Analysis of Non- Orthogonal Multiple Access for Visible Light Communications. China Commun..

[53] Yin L., Wu X., Haas H. On the Performance of Non-Orthogonal Multiple Access in Visible Light Communication. Proceedings of the 2015 IEEE 26th Annual International Symposium on Personal, Indoor, and Mobile Radio Communications (PIMRC).

[54] Lin B., Tang X., Ghassemlooy Z., Lin C., Zhang M., Zhou Z., Wu Y., Li H. A NOMA Scheme for Visible Light Communications Using a Single Carrier Transmission. Proceedings of the 2017 First South American Colloquium on Visible Light Communications (SACVLC).

[55] Wang G., Shao Y., Chen L.-K., Zhao J. (2021). Subcarrier and Power Allocation in OFDM-NOMA VLC Systems. IEEE Photon. Technol. Lett..

[56] Marshoud H., Sofotasios P.C., Muhaidat S., Karagiannidis G.K., Sharif B.S. Error Performance of NOMA VLC Systems. Proceedings of the 2017 IEEE International Conference on Communications (ICC).

[57] Chen C., Zhong W.-D., Yang H., Du P. (2018). On the Performance of MIMO-NOMA-Based Visible Light Communication Systems. IEEE Photon. Technol. Lett..

[58] Zhao Q., Jiang J., Wang Y., Du J. (2020). A Low Complexity Power Allocation Scheme for NOMA-Based Indoor VLC Systems. Opt. Commun..

[59] Yang Z., Xu W., Li Y. (2016). Fair Non-Orthogonal Multiple Access for Visible Light Communication Downlinks. IEEE Wirel. Commun. Lett..

[60] Zhang X., Gao Q., Gong C., Xu Z. (2017). User Grouping and Power Allocation for NOMA Visible Light Communication Multi-Cell Networks. IEEE Commun. Lett..

[61] Shen H., Wu Y., Xu W., Zhao C. (2017). Optimal Power Allocation for Downlink Two-User Non-Orthogonal Multiple Access in Visible Light Communication. J. Commun. Inf. Netw..

[62] Fu Y., Hong Y., Chen L.-K., Sung C.W. (2018). Enhanced Power Allocation for Sum Rate Maximization in OFDM-NOMA VLC Systems. IEEE Photon. Technol. Lett..

[63] Ma S., He Y., Li H., Lu S., Zhang F., Li S. (2019). Optimal Power Allocation for Mobile Users in Non-Orthogonal Multiple Access Visible Light Communication Networks. IEEE Trans. Commun..

[64] Dong Z., Shang T., Li Q., Tang T. (2019). Adaptive Power Allocation Scheme for Mobile NOMA Visible Light Communication System. Electronics.

[65] Feng S., Bai T., Hanzo L. (2019). Joint Power Allocation for the Multi-User NOMA-Downlink in a Power-Line-Fed VLC Network. IEEE Trans. Veh. Technol..

[66] Li Q., Shang T., Tang T., Dong Z. (2019). Optimal Power Allocation Scheme Based on Multi-Factor Control in Indoor NOMA-VLC Systems. IEEE Access.

[67] Liu H., Zhu P., Chen Y., Huang M. (2020). Power Allocation for Downlink Hybrid Power Line and Visible Light Communication System. IEEE Access.

[68] Dong Z., Shang T., Li Q., Tang T. (2020). Differential Evolution-Based Optimal Power Allocation Scheme for NOMA-VLC Systems. Opt. Express.

[69] Armstrong J. (2009). OFDM for Optical Communications. J. Lightwave Technol..

[70] Shieh W., Djordjevic I. (2009). OFDM for Optical Communications.

[71] Dissanayake S.D., Armstrong J. (2013). Comparison of ACO-OFDM, DCO-OFDM and ADO-OFDM in IM/DD Systems. J. Lightwave Technol..

[72] Chu W., Dang J., Zhang Z., Wu L. Effect of Clipping on the Achievable Rate of Non-Orthogonal Multiple Access with DCO-OFDM. Proceedings of the 2017 9th International Conference on Wireless Communications and Signal Processing (WCSP).

[73] Li H., Huang Z., Xiao Y., Zhan S., Ji Y. (2019). A Power and Spectrum Efficient NOMA Scheme for VLC Network Based on Hierarchical Pre-Distorted LACO-OFDM. IEEE Access.

[74] Gebeyehu Z.H. (2020). Impact of Clipping Noise on the Sum Rate of NOMA with PD-DCO-OFDM and Conventional DCO-OFDM. Heliyon.

[75] Jha M.K., Kumar N., Lakshmi Y.V.S. Performance of Zero-Biased NOMA VLC System. Proceedings of the 2020 IEEE 3rd 5G World Forum (5GWF).

[76] Fath T., Haas H. (2013). Performance Comparison of MIMO Techniques for Optical Wireless Communications in Indoor Environments. IEEE Trans. Commun..

[77] Gupta A.K., Chockalingam A. (2018). Performance of MIMO Modulation Schemes with Imaging Receivers in Visible Light Communication. J. Lightwave Technol..

[78] Rajput V.S., Ashok D.R., Chockalingam A. MU-MIMO NOMA with Linear Precoding Techniques in Indoor Downlink VLC Systems. Proceedings of the 2020 IEEE 91st Vehicular Technology Conference (VTC2020-Spring).

[79] Chen C., Yang Y., Deng X., Du P., Yang H., Chen Z., Zhong W.-D. NOMA for MIMO Visible Light Communications: A Spatial Domain Perspective. Proceedings of the 2019 IEEE Global Communications Conference (GLOBECOM).

[80] Marshoud H., Dawoud D., Kapinas V.M., Karagiannidis G.K., Muhaidat S., Sharif B. MU-MIMO Precoding for VLC with Imperfect CSI. Proceedings of the 2015 4th International Workshop on Optical Wireless Communications (IWOW).

[81] Dixit V., Kumar A. (2021). An Exact Error Analysis of Multi-User RC/MRC Based MIMO-NOMA-VLC System with Imperfect SIC. IEEE Access.

[82] Lin B., Ghassemlooy Z., Tang X., Li Y., Zhang M. (2017). Experimental Demonstration of Optical MIMO NOMA-VLC with Single Carrier Transmission. Opt. Commun..

[83] Shi J., Hong Y., He J., Deng R., Chen L.-K. Experimental Demonstration of OQAM-OFDM Based MIMO-NOMA over Visible Light Communications. Proceedings of the 2018 Optical Fiber Communication Conference (OFC).

[84] Wang H., Wang F., Li R. (2020). Enhancing Power Allocation Efficiency of NOMA Aided-MIMO Downlink VLC Networks. Opt. Commun..

[85] Liu X., Yu H., Zhu Y., Zhang E. Power Allocation Algorithm of Optical MIMO NOMA Visible Light Communications. Proceedings of the 2019 IEEE 9th International Conference on Electronics Information and Emergency Communication (ICEIEC).

[86] Mishra A.K., Trivedi A. Performance Analysis of MIMO-NOMA-Based Indoor Visible Light Communication in Single Reflection Environment. Proceedings of the 2019 IEEE Conference on Information and Communication Technology.

[87] Raj R., Dixit A. Performance Evaluation of Power Allocation Schemes for Non-Orthogonal Multiple Access in MIMO Visible Light Communication Links. Proceedings of the 2020 International Conference on Signal Processing and Communications (SPCOM).

[88] Jha M.K., Kumar N., Lakshmi Y.V.S. NOMA MIMO Visible Light Communication with ZF-SIC and MMSE-SIC. Proceedings of the 2020 2nd PhD Colloquium on Ethically Driven Innovation and Technology for Society (PhD EDITS).

[89] Ma S., Dong Z.-L., Li H., Lu Z., Li S. (2016). Optimal and Robust Secure Beamformer for Indoor MISO Visible Light Communication. J. Lightwave Technol..

[90] Du C., Zhang F., Ma S., Tang Y., Li H., Wang H., Li S. (2019). Secure Transmission for Downlink NOMA Visible Light Communication Networks. IEEE Access.

[91] Massey J.L. (1988). An Introduction to Contemporary Cryptology. Proc. IEEE.

[92] Zheng B., Wen M., Wang C.-X., Wang X., Chen F., Tang J., Ji F. (2018). Secure NOMA Based Two-Way Relay Networks Using Artificial Noise and Full Duplex. IEEE J. Sel. Areas Commun..

[93] Yesilkaya A., Cogalan T., Erkucuk S., Sadi Y., Panayirci E., Haas H., Poor H.V. Physical-Layer Security in Visible Light Communications. Proceedings of the 2020 2nd 6G Wireless Summit (6G SUMMIT).

[94] Wyner A.D. (1975). The Wire-Tap Channel. Bell Syst. Tech. J..

[95] Li Z., Trappe W., Yates R. Secret Communication via Multi-Antenna Transmission. Proceedings of the 2007 41st Annual Conference on Information Sciences and Systems.

[96] Liu X., Chen Z., Wang Y., Zhou F., Ma S. (2020). Robust Artificial Noise-Aided Beamforming for a Secure MISO-NOMA Visible Light Communication System. China Commun..

[97] Liu X., Wang Y., Zhou F., Ma S., Hu R.Q., Ng D.W.K. (2020). Beamforming Design for Secure MISO Visible Light Communication Networks With SLIPT. IEEE Trans. Commun..

[98] Zhao X., Chen H., Sun J. (2018). On Physical-Layer Security in Multiuser Visible Light Communication Systems with Non-Orthogonal Multiple Access. IEEE Access.

[99] Zhao X., Sun J. (2019). On Secrecy Performance of the Strong User in MISO-NOMA Visible Light Communication System. Electronics.

[100] Yang Y., Chen C., Zhang W., Deng X., Du P., Yang H., Zhong W.-D., Chen L. (2018). Secure and Private NOMA VLC Using OFDM with Two-Level Chaotic Encryption. Opt. Express.

[101] CISCO (2017). Cisco Visual Networking Index: Forecast and Methodology 2016–2021.

[102] Zvanovec S., Chvojka P., Haigh P. (2015). Visible Light Communications towards 5G. Radioengineering.

[103] Chi N., Haas H., Kavehrad M., Little T.D.C., Huang X.-L. (2015). Visible Light Communications: Demand Factors, Benefits and Opportunities [Guest Editorial]. IEEE Wirel. Commun..

[104] Rahaim M.B., Vegni A.M., Little T.D.C. A Hybrid Radio Frequency and Broadcast Visible Light Communication System. Proceedings of the 2011 IEEE GLOBECOM Workshops (GC Wkshps).

[105] Obeed M., Salhab A.M., Zummo S.A., Alouini M.-S. (2018). Joint Optimization of Power Allocation and Load Balancing for Hybrid VLC/RF Networks. J. Opt. Commun. Netw..

[106] Han Y., Zhou X., Yang L., Li S. A Bipartite Matching Based User Pairing Scheme for Hybrid VLC-RF NOMA Systems. Proceedings of the 2018 International Conference on Computing, Networking and Communications (ICNC).

[107] Zhou X., Li S., Zhang H., Wen Y., Han Y., Yuan D. Cooperative NOMA Based VLC/RF System with Simultaneous Wireless Information and Power Transfer. Proceedings of the 2018 IEEE/CIC International Conference on Communications in China (ICCC).

[108] Papanikolaou V.K., Diamantoulakis P.D., Ding Z., Muhaidat S., Karagiannidis G.K. Hybrid VLC/RF Networks with Non-Orthogonal Multiple Access. Proceedings of the 2018 IEEE Global Communications Conference (GLOBECOM).

[109] Papanikolaou V.K., Diamantoulakis P.D., Karagiannidis G.K. (2019). User Grouping for Hybrid VLC/RF Networks with NOMA: A Coalitional Game Approach. IEEE Access.

[110] Al Hammadi A., Muhaidat S., Sofotasios P.C., Al Qutayri M. A Robust and Energy Efficient NOMA-Enabled Hybrid VLC/RF Wireless Network. Proceedings of the 2019 IEEE Wireless Communications and Networking Conference (WCNC).

[111] Al Hammadi A., Sofotasios P.C., Muhaidat S., Al-Qutayri M., Elgala H. (2021). Non-Orthogonal Multiple Access for Hybrid VLC-RF Networks With Imperfect Channel State Information. IEEE Trans. Veh. Technol..

[112] Xiao Y., Diamantoulakis P.D., Fang Z., Ma Z., Hao L., Karagiannidis G.K. (2020). Hybrid Lightwave/RF Cooperative NOMA Networks. IEEE Trans. Wirel. Commun..

[113] Obeed M., Dahrouj H., Salhab A.M., Chaaban A., Zummo S.A., Alouini M.-S. (2021). Power Allocation and Link Selection for Multicell Cooperative NOMA Hybrid VLC/RF Systems. IEEE Commun. Lett..

[114] Obeed M., Dahrouj H., Salhab A.M., Zummo S.A., Alouini M.-S. (2021). User Pairing, Link Selection, and Power Allocation for Cooperative NOMA Hybrid VLC/RF Systems. IEEE Trans. Wirel. Commun..

[115] Raj R., Dixit A. (2021). Outage Analysis and Reliability Enhancement of Hybrid VLC-RF Networks Using Cooperative Non-Orthogonal Multiple Access. IEEE Trans. Netw. Serv. Manag..

[116] Guan X., Hong Y., Yang Q., Chan C.C.-K. Phase Pre-Distortion for Non-Orthogonal Multiple Access in Visible Light Communications. Proceedings of the Optical Fiber Communication Conference.

[117] Guan X., Yang Q., Chan C.-K. (2017). Joint Detection of Visible Light Communication Signals Under Non-Orthogonal Multiple Access. IEEE Photon. Technol. Lett..

[118] Lin B., Lai Q., Ghassemlooy Z., Tang X. (2021). A Machine Learning Based Signal Demodulator in NOMA-VLC. J. Lightwave Technol..

[119] Li H., Huang Z., Xiao Y., Zhan S., Ji Y. (2017). Solution for Error Propagation in a NOMA-Based VLC Network: Symmetric Superposition Coding. Opt. Express.

[120] Mounir M., El_Mashade M.B., Mohamed Aboshosha A. (2021). On The Selection of Power Allocation Strategy in Power Domain Non-Orthogonal Multiple Access (PD-NOMA) for 6G and Beyond. Trans. Emerg. Telecommun. Technol..

[121] Huang Y., Wang J., Jianyue Z. (2019). Optimal Power Allocation for Downlink NOMA Systems. Multiple Access Techniques for 5G Wireless Networks and Beyond.

